# Patient radiation dosimetry for fluoroscopically guided interventions – a review of the history and limitations

**DOI:** 10.1002/mp.70211

**Published:** 2026-01-05

**Authors:** Kevin A. Wunderle, Stephen Balter

**Affiliations:** ^1^ Division of Medical Physics Department of Radiology College of Medicine The Ohio State University Wexner Medical Center Columbus Ohio USA; ^2^ Departments of Radiology and Medicine The Columbia University Medical Center New York New York USA

**Keywords:** dosimetry, fluoroscopically guided interventions (FGI)

## Abstract

Fluoroscopically guided interventions (FGIs) play a critical role in modern medicine, though they potentially deliver high radiation doses that require accurate and patient‐specific dosimetry for risk assessment and patient management. This review provides a historical overview of radiation dosimetry in fluoroscopy, tracing the evolution from rudimentary dose estimation methods to modern tools leveraging standardized dose indices and radiation dose structured reports (RDSRs) for radiation dosimetry at the skin surface as well as at depth in soft tissue. Despite these advancements, substantial uncertainties remain due to technical limitations, beam geometry, dose mapping challenges, and hardware‐induced variability. Key sources of uncertainty are examined, some well‐known and others that are not well‐understood. This work also underscores the need for a deeper understanding of the assumptions and limitations in automated dosimetry systems and calls for enhanced reporting standards and dose mapping capabilities to improve accuracy. Addressing these limitations is essential for integrating radiobiological principles into clinical medical physics and optimizing patient care in FGIs.

## 1 INTRODUCTION

Understanding historical aspects of fluoroscopy as an imaging modality is essential for appreciating advances in state‐of‐the‐art equipment and related radiation dosimetry. The historical context may also help clarify limitations by definition or design, which may not otherwise make sense given technological advances. Over the past three decades, innovations in hardware and data technologies have substantially enhanced the monitoring and recording of technical parameters, enabling radiation dose estimation and patient‐specific dose distributions previously unattainable. These advances have drastically improved dosimetry for fluoroscopically guided interventions (FGI) and allowed numerous automated or semi‐automated approaches to estimating patient radiation dose and dose distributions.^1^, ^2^, ^3^, ^4^, ^5^

However, despite considerable advancements, many limitations and uncertainties remain in FGI patient dosimetry. These challenges are often overlooked, particularly with the increased reliance on automated software‐based approaches. These limitations and uncertainties should be understood by medical physicists using these data for patient dosimetry or clinical procedure analysis. Such limitations might impact clinical care or clinical practice in three increasingly important ways. First either by necessitating excessive conservatism, potentially leading to unnecessary patient follow‐ups, or by producing overly liberal dosimetry estimates, which may fail to identify patients at risk of moderate to severe tissue reactions. Second, accurate radiation dose indices are necessary for establishing quality diagnostic reference level (DRL) values for FGI procedures, such as those hopefully to come from the American College of Radiology's Fluoroscopy Dose Index Registry (ACR Fluoro DIR).^6^, ^7^, ^8^ DRL values may prompt clinical teams to adjust their imaging systems to better align to published distributions. However, without reasonably accurate doses or dose indices, aggregated clinical data distributions are subject to greater uncertainty, decreasing their utility or potentially yielding misguiding results. Third, with expanding and advancing interventional clinical practice and imaging capabilities, particularly in interventional oncology, there are FGI patients that undergo multiple high dose procedures and some that even cross over clinical service to also receive external beam radiotherapy.^9^ These cases present a complex radiation dosimetry and radiobiology challenge with the need for better radiation dosimetry accuracy and precision.

This article aims to review historical and current fluoroscope design, definitions, and limitations related to radiation dosimetry, both in determining entrance kerma (K_a,e_) and radiation dose deposition in water. Radiation deposition to other tissues, in particular bone, is also vitally important for patient outcomes, as is an understanding of radiogenic tissue reactions, but these topics are outside the scope of this article and are intended to be covered in a second review article to follow, specifically addressing these topics.

### 1.1 FGI dosimetry—historical background and modern advances

From the 1960s to the 1990s, real‐time dynamic images (fluoroscopy mode imaging) were viewed via image intensifiers and analog video chains. Recording single images or dynamic image sequences (acquisition mode imaging) were either accomplished via optical cameras (e.g., photospot or cine) focused on the image intensifier output phosphor, or by an external film‐changer.^10^, ^11^

The regulations and standards pertaining to fluoroscopy as an imaging modality were established when the distinctions between these modes of imaging were self‐evident. However, the transition to digital image receptors and real‐time digital image capture has blurred these differences which now exist primarily due to their historical origins and the regulatory framework developed around them, which has remained largely unchanged for decades.

The fluoroscopy and acquisition modes of operation have markedly different radiation outputs and effects on patient dosimetry, with acquisitions often contributing a substantial fraction of the total dose in many FGI procedures.^12^ In the era of analog image receptors, imposing dose rate limits on fluoroscopy mode imaging while excluding such restrictions in acquisition mode imaging was logical, because film inherently constrained dose levels for a clinically acceptable image. With modern digital image receptors, however, image quality generally does not degrade with increasing dose or dose rates, and, in the absence of regulatory limits, doses may unknowingly surpass clinical necessity. This technological shift underscores that regulatory and standards frameworks, developed decades ago for very different technology, may no longer be sufficient and should be periodically reevaluated. Continuing to use decades old conventions may be detrimental, either artificially constraining the modality from technological advancement or conversely allowing excessively high radiation dose rates where they may not be necessary. The continued use of potentially outdated traditional fluoroscopic air kerma rate limits, while acquisition modes remain without limits, highlights a critical gap, but an opportunity. Moving forward, organizations that produce or maintain regulations, standards, and best practices should substantiate dose rate limits and operating mode restrictions so that they appropriately reflect contemporary technological capabilities and clinical image quality demands, rather than relying on antiquated rationales.

Before the mid‐2000s, patient radiation dosimetry was often derived using the procedure fluoroscopy beam‐on time, the number and length of serial radiographic series (or number of radiographic frames acquired), and some estimate of a patient's habitus to allow comparison with surrogate phantom dosimetric data. Patient dose estimates were at best crude due to unknown parameters such as the geometric positioning of the fluoroscope gantry and patient, radiation beam quality, radiation beam area and shape, and patient habitus, amongst many others.

Prospective direct superficial radiation dosimetry for FGI procedures has been effectively performed using several methods. Historically, methods relied on X‐ray films and thermoluminescent dosimeters (TLDs). More recent capabilities include optically stimulated luminescent dosimeters (OSLDs) and self‐developing radiochromic films.^13^, ^14^ The measurement media are placed directly on or near the patient's skin to allow for direct measurement of the entrance air kerma (K_a,e_). Films offer the additional benefit of acquiring a two‐dimensional distribution of K_a,e_ over the covered area, providing valuable information about the dose heterogeneity across the skin surface.^13^, ^14^

Regardless of the type, all direct dosimeters require some form of calibration and processing to ensure accuracy. Calibration is necessary over a wide range of relevant radiation doses and, potentially, across various X‐ray beam qualities used in clinical settings. This calibration process is generally applicable only to a specific batch (lot) of dosimeters or film, requiring recalibration whenever new batches are acquired. These mandatory calibration and processing steps can be labor‐intensive and time‐consuming, which has historically limited their routine clinical use for real‐time patient monitoring.^15^

For the direct assessment of internal organ doses, researchers have employed anthropomorphic phantoms, physical models that represent a “standard” human body. Dosimeters, such as TLDs, are strategically inserted into the anatomical locations of specific organs within the phantom. The phantom is then exposed to radiation protocols that mimic actual clinical procedures, and the TLDs are subsequently read to provide their respective organ doses. This method provides essential data for establishing dose reference levels and also for validating fully computational dose models such as those performed by Monte Carlo techniques.^14^, ^16^

Two substantial changes occurred in the early‐ to mid‐ 2000s. First, new fluoroscopes were required to report the radiation dose indices air kerma rate (${\dot{K}}_{a,r}$) during imaging and the cumulative air kerma ($K_{a,r}$) at the patient entrance reference point (PERP) by International Electrotechnical Commission (IEC) standard and subsequently the United States Food and Drug Administration (FDA).^17^, ^18^, ^19^ Second, the digital imaging and communications in medicine (DICOM) organization created a standard for interventional fluoroscopes providing the framework for radiation dose structured reports (RDSR) to be created and accessible to end users, which was based on an IEC Publicly Available Specification.^20^, ^21^ The RDSR standard required numerous geometric and technical factors to be recorded for every irradiation event during a procedure which was then compiled into a single record.^22^, ^23^ These two requirements greatly improved the availability and accuracy of technical information needed to estimate patient doses allowing medical physicists access to previously unavailable information and the capability to substantially refine patient dosimetry.

Current approaches to patient dosimetry from FGI procedures range from various manual calculations based on published methods from more than a decade ago,^24^, ^25^ to fully automated approaches by fluoroscope manufacturers in real‐time which track the patient positioning and map the dose distribution.^3^ There are also numerous third party radiation dose monitoring systems and standalone fluoroscopy dose mapping programs, including some freely available, that will digest an RDSR and provide some version of an estimated dose or dose distribution, accounting for various factors, with varying degrees of accuracy.^5^, ^26^

### 1.2 Radiation measurement devices

A critical aspect of any discussion regarding radiation dosimetry is the calibration and proper use of the system measuring or calculating the dose quantities. The fluoroscopic dose indices, provided by the fluoroscopic system, are calibrated or verified by service engineers or medical physicists using external dosimeters. The accuracy of these external devices directly affects the achievable accuracy of the systems they calibrate. The external dosimeters are either ionization chambers or, more commonly today, solid‐state detectors. Each type of dosimeter has its own advantages and potential challenges, depending on the specific application.

External ionization chambers used to measure fluoroscopic output, or to calibrate fluoroscopic systems, are generally classified as either reference‐class meters or field‐class meters. Ionization chambers are the gold standard as their response over a wide range of beam quality is relatively flat and well known.^27^, ^28^ Reference‐class meters must meet the IEC standard for combined uncertainty within ± 15%, while field‐class meters are permitted a higher uncertainty, up to ± 25%.^29^ It is expected, however, that devices used by clinical physicists or engineers to calibrate fluoroscopes should achieve substantially better accuracy than ± 15%. Depending on the calibration and the beam quality being evaluated, external ionization chambers can potentially achieve an uncertainty of ± 5%.^6^, ^27^, ^28^

Solid‐state dose or multi‐purpose detectors have markedly improved over the past two decades. They are now widely used by clinical physicists and field service engineers due to their efficiency in collecting and analyzing radiation exposure and beam quality data. However, caution must be exercised to ensure these devices are used as designed and intended, otherwise erroneous results may be obtained. For example, some solid‐state detectors contain lead or other highly attenuating material in their housing which may directly affect the output of the fluoroscope. Additionally, some solid‐state detectors require a specific orientation with respect to the X‐ray beam to avoid effects of the anode heel effect on the measurement. Solid‐state detectors often demonstrate a substantial energy dependence, mandating calibration for the range of X‐ray beam quality being evaluated. When used as intended and within their calibration conditions uncertainties of ±5% are commonly achieved.^6^, ^28^, ^30^, ^31^, ^32^

Everything that follows in this work assumes that the external dosimeters used to calibrate, establish, or evaluate the fluoroscope displayed dose indices, or to derive correction factors for the displayed dose indices, are appropriate, used correctly, and calibrated for the beam quality evaluated.

### 1.3 FGI dose

When referring to ‘dose’ or ‘patient dose’ in the context of FGIs, clarity is essential, as these terms can refer to various radiation quantities with substantially different meanings and implications. Differences in understanding or use of the term “dose” by different clinical professionals can also lead to miscommunication and confusion. Even within the context of fluoroscopic irradiation the term “dose” may be used in reference to various radiation quantities and indices, such as, the K_a,r_, the air kerma‐area product (P_KA_), the peak skin dose (PSD), a skin absorbed dose map, the effective dose (*E*), among others. K_a,r_ and P_KA_ are often referred to by clinicians as a ‘patient dose’ from a given FGI, however, both quantities are dose indices, and, though they may be useful and relate to a patient dose, they are not patient doses in and of themselves. The primary patient doses of interest from a fluoroscopic procedure are the *E* and, for some FGIs, the highest absorbed dose(s) to some area(s) of the skin, commonly referred to as the PSD, or potentially other organs, either as a discrete value or in the form of an absorbed dose map.

The derived quantity *E* is a source of considerable confusion, as it is frequently misinterpreted as a directly measurable patient radiation quantity rather than a calculated estimate of stochastic risk. In general, *E* estimates are calculated from empirically derived conversion factors acquired from anthropomorphic phantom studies, or Monte Carlo simulations, that are used to convert an imparted energy (typically P_KA_) to *E* given an exposed anatomic region, hence known organs exposed.^33^, ^34^, ^35^, ^36^, ^37^ As such, many of the uncertainties discussed in the present work apply to the starting point (P_KA_) for deriving *E*. Furthermore, *E* is not intended as an individual's risk but rather an estimate of risk to a standard person.^38^ Estimates of *E*s for FGIs are generally well below 100 mSv, however, they can exceed 100 mSv in some cases—though *E*s of that magnitude are typically encountered only from procedures on high acuity patients, often when surgical intervention is not possible.^14^, ^36^ Although *E* may be appreciable in some FGI procedures, the carcinogenic risk is generally substantially outweighed by the benefit of the procedure to the patient.^39^ Therefore, given the relatively straightforward approaches used to derive *E* from FGIs, the lack of patient specific applications of *E*, and the assumed clear benefit to the patient from FGI procedures, *E* will not be discussed further in this article.

Organ absorbed dose approximations, most commonly skin, can be computationally estimated using dose indices and other technical information provided by fluoroscopes as a starting point. However, accurate organ dose estimates are complicated by the fact that only a fraction of a given organ is typically irradiated, and that fraction is irradiated non‐uniformly. In the United States, the dose index most commonly available as a starting point for organ dose estimation is the K_a,r_ since it is required to be provided by various standards or regulatory bodies.^17^, ^40^, ^41^ Although it can be used as a starting point for a skin dose, K_a,r_ does not account for any geometric variables, including the fluoroscope c‐arm gantry orientation, position or motion; the patient orientation, position, or motion; or the procedure table orientation, position, or motion relative to the radiation field. The first step to deriving a patient related dose is to determine the incident air kerma (K_a,i_) to some surface area of the patient. To reach a K_a,i_, geometric corrections must be applied to the K_a,r_ for those variables, which can typically be accomplished by use of RDSR irradiation event level data and some mathematical model of the patient.^5^

Once the K_a,i_ to some area of interest is known, the skin absorbed dose can be derived based on published approaches.^12^, ^25^, ^42^

$$\begin{array}{cc} & \mathrm{Skin}\;\mathrm{Entrance}\;\mathrm{Absorbed}\;\mathrm{Dose}\;\\ & \;=K_{a,i}\;x\;BSF\;x\;f_{\mathrm{soft}\;\mathrm{tissue}}\;x\;CF_{\mathrm{Table}}\;x\;CF_{KA} \end{array}$$

where K_a,i_ is the incident air kerma to an area of tissue; BSF is the back scatter factor which is dependent on X‐ray beam half‐value layer (HVL), the size of the area irradiated, and the effective atomic number of the tissue exposed (typically skin for FGI procedures); the f‐factor is the correction factor applied to account for the difference between air kerma and absorbed dose for the tissue in question, which, in the present usage is generally taken as 1.06 for soft‐tissue, but may be as high as 4 for bone at diagnostic X‐ray energies; CF_Table_ is the correction factor for the presence of the procedure table and mattress (applied to radiation event level data when the table and mattress provide pre‐patient attenuation); and CF_KA_ is the correction factor to account for the accuracy of the fluoroscope provided air kerma related dose indices.^1^, ^43^, ^44^, ^45^, ^46^, ^47^ Additionally, for organ doses at depth the use of a Percent PDD or Tisue Air Ratio (TAR) values must be used to modify the skin entrance dose or incident air kerma, respectively.

## 2 K_a,r_ and P_KA_—ACCURACY and HARDWARE EFFECTS

Patient dose calculations rely on fluoroscope reported dose indices, which have inherent complexities, some that are well understood and often accounted for, and others that are not. This section focuses on variables and uncertainties associated with the reported K_a,r_ and P_KA_, which have implications for determining patient radiation doses. While some of these variables have been previously reported and discussed in literature, others are often overlooked or not well understood. Nonetheless, all may influence the accuracy of a derived skin entrance dose or tissue dose at depth when calculated from a fluoroscope‐reported K_a,r_.^1^

First, fluoroscope manufacturers use various methods to obtain the displayed dose indices, K_a,r_ and P_KA_. The two quantities are linked by the X‐ray field size at the patient entrance reference point (PERP), and systems differ in which quantity is measured, and which is derived. One common method is to measure the P_KA_ directly with a transmission ionization chamber and then derive the K_a,r_ by dividing the P_KA_ by the field size. Conversely, many newer systems estimate the K_a,r_ from look‐up tables based on technique factors and filtration; this estimate is then multiplied by the field size to derive the P_KA_. In both approaches, the field size is rarely a direct measurement but is instead estimated from the X‐ray beam collimator blade positions. While some very specific types of measurement devices can directly measure both quantities, they are rarely utilized.e

Although requirements for fluoroscopes to determine and report radiation dose indices have markedly improved patient dose estimation, large potential uncertainties remain. One of the largest uncertainties stems from the FDA regulations and IEC standards allowing for a ± 35% tolerance on the displayed and reported radiation dose indices.^17^, ^18^, ^48^

When these dose indices were first incorporated into the IEC standards, radiation output was typically measured using a transmission ionization chamber placed in the collimator assembly or housing. At the time, IECs standards permitted a measurement uncertainty of ± 25% for the measurement device alone.^29^ To account for system integration factors, an additional ± 10% margin was included in the imaging equipment standards, resulting in the ± 35% tolerance still specified in the current standard.^40^ These tolerances introduce potentially large uncertainties between fluoroscopic systems within and between institutions while still meeting regulatory requirements. Modern systems are certainly capable of achieving and maintaining much better accuracy, as indicated by previous publications^6^, ^49^ it seems an appropriate time for the regulations and standards to consider tightening these tolerances.

Measurement devices and software‐based dose estimation methods integrated into imaging equipment or third‐party systems should account for this uncertainty by applying user‐derived correction factors that are periodically updated to the indicated or reported values. The American Association of Physicists in Medicine (AAPM) Report 190 provides a methodology for determining correction factors for reported dose indices.^50^ Applying these correction factors may substantially improve the accuracy of the machine‐reported dose indices; however, using a single correction factor determined at a specific beam quality across a broad range of qualities may still result in approximately 15% uncertainty, depending on beam quality difference relative to calibration conditions.^51^ Establishing multiple correction factors across a wide range of beam qualities, particularly when pediatric procedures are considered, can further reduce uncertainty.

The accuracy of displayed dose indices should be assessed by a qualified medical physicist (QMP) during acceptance testing, at least annually thereafter, and following any repairs that could affect these values.^52^, ^53^, ^54^ Not only should the required tolerances be verified, but the accuracy should be accounted for when performing any type of radiation dose estimation, or when comparing aggregated radiation doses with reference data sets. Additionally, there are several fluoroscope hardware related factors that can contribute substantial uncertainty in patient dose estimates.

### 2.1 Table and mattress attenuation

Patient procedure tables and mattresses provide pre‐patient X‐ray beam attenuation and scatter in posteroanterior (PA) projections. As the C‐arm gantry rotates away from the PA position around the patient, the fraction of the beam attenuated by the table and pad decreases. This attenuation is ultimately eliminated at primary rotational angles beyond lateral and approaching anteroposterior (AP) projections. This variability in pre‐patient attenuation is not accounted for in the fluoroscope reported K_a,r_ or P_KA_.^55^

A single correction factor is typically used to account for the attenuation effects of the table and mattress. This factor is generally determined based on a fixed X‐ray beam quality and field size under normal incidence. However, substantial variability exists in the transmission fraction across the area of the X‐ray beam due to path length differences and beam quality dependencies across the range of energies encountered. Reported correction factors range from 0.52 to 0.83 across a wide range of beam qualities, with additional path‐length corrections needed for non‐normal beam incidence.^46^, ^55^

As the C‐arm gantry rotates through primary angles (right anterior oblique (RAO) and left anterior oblique (LAO)) or secondary angles (cranial (CRA) and caudal (CAU)), portions of the X‐ray beam will directly reach the patient's skin entrance, while others will be pre‐attenuated by the table and mattress at steep angles. The angles at which the transition occurs will depend on several factors: the patient's axial dimensions; the orientation and positioning of the patient, procedure table, and C‐arm gantry; and the X‐ray beam field size.

### 2.2 Collimation (field size/shape)

The size and shape of the X‐ray field introduce additional variability in dose estimation. Proper collimation is essential for limiting radiation exposure to the anatomic region of interest, thereby reducing unnecessary dose to surrounding tissues. However, the RDSR does not consistently record the exact size and shape of the X‐ray field.

For archived images, pixel information may be used to estimate the X‐ray field size and shape. However, not all procedures have archived images, and even when images are available, most radiation events within a procedure will not have recorded images.^43^ Furthermore, most estimations assume that the X‐ray field is confined to the active area of the image receptor, which is not always the case and is allowed to exceed the image receptor area by amounts specified by regulation.

A rough approximation of the X‐ray beam area can be obtained at the irradiation event level by dividing the P_KA_ by K_a,r,_ yielding an estimated area in the plane of the PERP. However, this method does not provide information about the shape of the X‐ray field, which is critical for evaluating field overlap and dose mapping. The resulting deviations in dose estimates will scale with changes in collimated beam area and shape.

The uncertainty in beam size and shape may potentially be addressed with the Patient RDSR (P‐RDSR)^56^ the next iteration of the RDSR, which proposes including both the area and shape of the X‐ray field. Implementing P‐RDSR could substantially improve dose estimation accuracy and facilitate more precise dose mapping. But adoption of the P‐RDSR is likely to be slow and, since DICOM standards are voluntary, may not happen at all unless it becomes required by other standards or regulatory bodies.

### 2.3 Tissue equalization filters

The use of tissue equalization filters (also known as wedge or tissue compensation filters) is considered best practice, particularly in anatomic regions such the heart where more radio‐opaque tissue (cardiac muscle) is near radio‐transparent tissue (lung) which can result in image burnout.^10^ These filters help compensate for large variations in tissue attenuation and equalize the photon flux across the image receptor, providing better gray scale mapping, and displayed image quality. In some cases, these filters are automatically pre‐positioned as part of specific fluoroscopic imaging protocols (such as cardiac) but can always be manipulated around the image field or removed from the image field all together.

However, incorporating the effects of these filters on dose estimates is quite challenging. Their position or even use are not typically recorded in RDSRs, and even if they were, modeling their impact would be difficult. The filters are often wedge‐shaped by design resulting in an X‐ray flux gradient that can be oriented in numerous positions within the X‐ray field. Consequently, local tissue doses within the X‐ray field may vary substantially, with dose varying 50%, or more, in regions affected by these filters and by the method of calculation used.

## 3 X‐RAY FIELD INHOMOGENEITY, DEPTH DOSE, and LATERAL SPREAD

### 3.1 X‐ray beam quality

X‐ray beam quality affects all aspects of dosimetry, it directly affects the skin dose, as indicated in the skin dose equation provided earlier, but also impacts both the dose distribution within a plane and the dose deposition at depth in tissue. In this section, dose deposition is discussed, and it is important to note that the Table 1 values were collected in water and therefore are relative values based on the absorbed dose to water, hence back scatter is included. Table 1 provides the radiation beam qualities measured from an interventional fluoroscope with a full range of spectral filtration, the same fluoroscope used to acquire the PDD data presented later. The data include the first and second half‐value layers, homogeneity coefficients, and backscatter factors for three nominal X‐ray fields of view at 60 cm from the focal spot.^47^ This data may provide useful starting points or reference data for patient radiation dosimetry or related quality assurance testing.

**TABLE 1:** HVLs, HCs, and BSFs for a full range of fluoroscopic X‐ray beam qualities. Table reproduced with permission from Wunderle et al. 2017.^47^

kVp	Cu (mm)	HVL1 (mm Al)	HVL2 (mm Al)	HC	Backscatter factors 11 cm FOV	Backscatter factors 22 cm FOV	Backscatter factors 42 cm FOV
60	0	2.19	3.00	0.73	1.18	1.28	1.35
60	0.1	3.41	4.07	0.84	1.21	1.34	1.44
60	0.3	4.65	5.28	0.88	1.23	1.39	1.51
60	0.6	5.61	6.06	0.93	1.24	1.41	1.54
60	0.9	6.25	6.49	0.96	1.23	1.41	1.56
80	0	2.89	4.31	0.67	1.19	1.32	1.40
80	0.1	4.56	5.95	0.77	1.21	1.37	1.49
80	0.3	6.33	7.21	0.88	1.23	1.40	1.55
80	0.6	7.62	8.26	0.92	1.23	1.41	1.58
80	0.9	8.35	8.96	0.93	1.23	1.42	1.58
100	0	3.62	5.78	0.63	1.19	1.33	1.44
100	0.1	5.61	7.45	0.75	1.21	1.38	1.51
100	0.3	7.61	8.80	0.86	1.22	1.39	1.55
100	0.6	8.90	9.73	0.92	1.22	1.40	1.57
100	0.9	9.83	10.29	0.96	1.21	1.39	1.56
120	0	4.37	7.09	0.62	1.20	1.34	1.45
120	0.1	6.55	8.39	0.78	1.21	1.37	1.51
120	0.3	8.56	9.79	0.87	1.21	1.38	1.54
120	0.6	9.95	10.85	0.92	1.20	1.38	1.54
120	0.9	10.82	11.62	0.93	1.20	1.37	1.54

### 3.2 Heel effect

Typically, K_a,r_ and any derived correction factors are determined along the central axis of the X‐ray beam. However, air kerma and beam quality are not uniform across the X‐ray beam cross‐sectional area due to the anode heel effect. This phenomenon was evident in screen film radiography as it was inherently present and generally visible in every image. With the transition to digital X‐ray imaging, flat field correction factors largely eliminate the visibility of the heel effect from processed digital images. Though not as observable, the phenomenon is still present and ignoring its effect on dosimetry may result in substantial errors due to the assumption of a uniform kerma across the area of the skin entrance.

**FIGURE 1:**
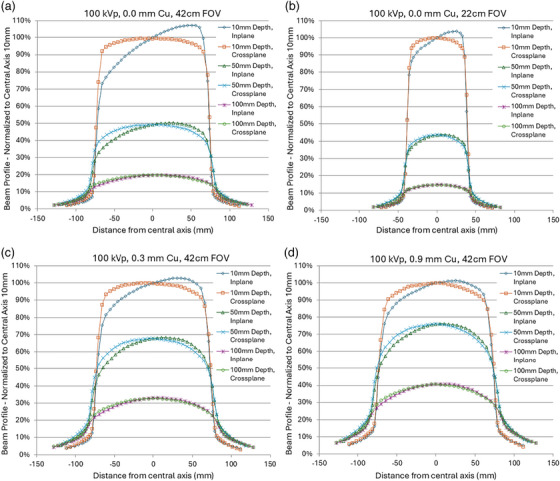
X‐ray beam profiles curves for a single fluoroscopic beam quality at two different fields of view illustrating the heel effect and how it changes with depth in water. (a) In‐plane and cross‐plane profile scans at three depths in water for the nominal 42 cm field of view. (b) In‐plane and cross‐plane profile scans at three depths in water phantom for the nominal 22 cm field of view. (c) In‐plane and cross‐plane profile scans at three depths in water for the nominal 42 cm field of view with 0.3 mm Cu spectral filtration. (d) In‐plane and cross‐plane profile scans at three depths in water for the nominal 42 cm field of view with 0.9 mm Cu spectral filtration. Figure adopted with permission from Wunderle et al 2019.^57^

For example, Figure 1 illustrates the absorbed dose in water at a depth of 1 cm for a large field, where the difference in dose between the cathode and anode ends of the field is approximately 35%, with potentially even greater variations at the surface or for large x‐ray fields.^57^ If air kerma or entrance kerma is measured at the center of the field, this variation is distributed, resulting in an uncertainty of approximately ± 15–20%, which is not negligible.

Despite its magnitude, this phenomenon is rarely, if ever, accounted for in dose calculations, including software‐based dose calculators and dose maps. Future dose mapping approaches may want to consider this source of error and model the effect in their dose calculations.

### 3.3 Percent depth dose (PDD)

Percent depth dose (PDD) is a fundamental concept in radiation physics and dosimetry that quantifies how radiation dose is deposited as a function of depth within a uniform medium, typically water or soft tissue. For kilovolt energy X‐rays used in diagnostic imaging PDDs play a crucial role in understanding radiation absorption in the human body and enables more accurate dosimetry at depth.

**FIGURE 2:**
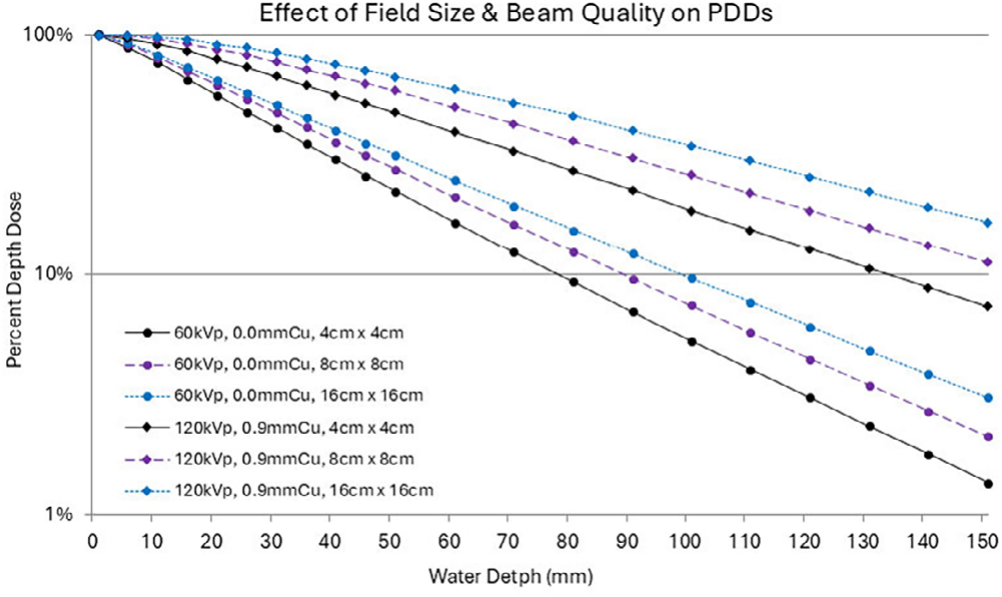
PDDs in water phantom measured for low and high beam quality for the nominal 11, 22, and 42 cm fields of view. at 60 cm source to focal spot distance. Figure adopted with permission from Wunderle et al 2017.^47^ PDDs, Percent depth dose.

As an X‐ray beam passes through a medium the processes of attenuation and scatter reduce photon and energy flux. Dose deposition at depth in a medium is affected by many variables, including the composition of the medium (density and effective atomic number), the X‐ray beam quality, and the X‐ray beam field size, among various other factors. There are a broad range of possible X‐ray beam qualities produced by state‐of‐the‐art interventional fluoroscopes given the range of commonly available kVps and availability of spectral filtration. The X‐ray beam quality for any given irradiation event is predicated on several factors; first, the automatic dose rate control logic, which is determined by the selected imaging protocol and dose curve, second, the water equivalent thickness of the object imaged, which is determined from the image receptor signal relative to the known output.^47^ Spectral filtration, most often Cu but other materials are used, substantially affect PDDs. Figure 2 is adapted from Wunderle et al 2017 and illustrates the effect of various amounts of Cu filtration on the PDDs in water for a given geometry, X‐ray beam field size, and kVp. PDDs are essential for determining the absorbed dose at depth within a tissue or organ and depend primarily on the X‐ray beam quality and beam area.

### 3.4 X‐ray beam profiles and lateral spread

Radiation dose to the skin surface is a primary focus of radiation dosimetry for FGIs. However, in isocentric imaging, where the anatomy of interest is positioned at the isocenter of the C‐arm geometry, it is important to recognize that all projections along the patient's *z*‐axis (head to foot) will overlap at some tissue depth. While discrete X‐ray beams at the skin surface may not overlap on the surface, they may still intersect at shallow tissue depths, irradiating a common tissue volume. Isocentric imaging is commonly employed in neurointerventions, cardiac interventions, and certain body interventions and will irradiate much of the same tissue volume, regardless of the specific geometries used. Even for non‐isocentric imaging techniques, when the same target anatomy is imaged, overlapping X‐ray fields are inevitable at some tissue depth.

**FIGURE 3:**
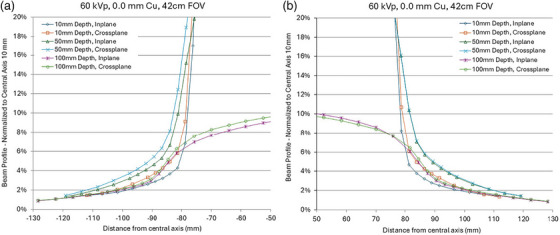
(a) Left, (b) Right, selected views of the various X‐ray beam profile tails. Figure adopted with permission from Wunderle et al 2019.^57^

As the primary X‐ray beam enters and penetrates tissue, geometric beam divergence and scattered X‐rays contribute to dose deposition beyond the boundaries of the primary X‐ray field at the skin surface with increasing depth. The dimensions of the primary X‐ray beam are typically characterized by Full Width at Half Maximum (FWHM) or Full Width at Tenth Maximum (FWTM) of the x‐ray beam in the medium. However, despite substantial attenuation, a portion of the dose extends beyond the primary field boundaries (see Figure 3). Several percent of the maximum dose deposited in water may be present outside the FWHM or FWTM fields, extending up to a few centimeters^57^ The extent of this dose deposition depends on multiple factors, including the nominal X‐ray field size, X‐ray beam quality, irradiated tissue composition, tissue surface geometry, and other variables.

As illustrated in Figure 3, lateral dose diffusion increases with tissue depth, reaching a peak at approximately 5 cm, due to contributions from forward, lateral, and backscatter, in addition to beam divergence. These dose contributions to the skin and adjacent organs are rarely, if ever, considered in standard dose calculations. As computational models become more advanced, incorporating these factors may be necessary to account for adjacent X‐ray field overlap near the surface as well as cumulative dose deposition at depth.

## 4 TOTAL UNCERTAINTY

Taken together, the uncertainties in non‐automated skin absorbed dose estimates by physicists using summary procedure level radiation dose data may be on the order ± 50%.^25^ With automated software approaches, this uncertainty is generally reduced due to the more refined methods which use irradiation event level data from RDSRs and reportedly yield an uncertainty of 20–30%.^1^, ^2^ However, those reported accuracies do not assume all of the possible uncertainties discussed herein, such as the inhomogeneity of X‐ray field intensity and beam quality due to the heel effect, use of tissue compensation (wedge) filters, X‐ray field size and shape uncertainty, among others. These total uncertainties also assume several other factors, first that the fluoroscope dose indices are within the regulatory tolerance (which may not be true and why they must be periodically assessed). Second, that the calculation has correctly registered or otherwise properly accounted for the location and positioning of the patient. In general, patients undergoing FGI procedures are sedated, either under conscious sedation or fully unconscious under general anesthesia, which would limit the amount of patient motion. But otherwise, patient positioning and the ability to account for any changes in that positioning may be a large source of uncertainty. Some efforts have been made to use external cameras or other monitoring devices to track the patient movement for registration purposes.^58^ Additionally, it is assumed that the X‐ray technique factors reported in the RDSR are accurate, the location and positioning of the procedure table and mattress are known and their effects on the X‐ray beam relative to the patient are accounted for, the device used to calibrate the fluoroscope was appropriate and calibrated for the beam quality measured during calibration, among other variables. Without these assumptions being valid, or wherever they are unknown, the uncertainty may increase well beyond 20%–50%.

## 5 DISCUSSION

Historically, reliance on simple metrics such as fluoroscopy beam‐on time and acquisition counts provided only crude estimates of patient dose, lacking the precision needed for individualized dosimetry and risk evaluation. Advances in the mid‐2000s, including the introduction of standardized radiation dose indices like K_a,r_ and the development of RDSRs, substantially improved the accuracy of dosimetric assessments. However, challenges remain due to uncertainties arising from technical factors such as variations in beam geometry, X‐ray beam nonuniformity, and attenuation caused by components in the beam path, such as tables and mattresses.

Potential clinical use of patient radiation dose estimates vary. Without any definitive sources describing current uses, there are several obvious current and potential future uses known to the authors. First, there are occasions when patients want to know their “radiation dose”, this situation is complicated as most patients don't understand the complexities in estimating radiation doses nor nuances of either *E* or absorbed tissue dose, though at times this is requested and necessary to provide. Second, radiation dose estimates are performed to assess the risk of a radiogenic tissue reaction. This may be done at the request of a physician performing an FGI procedure, the result of a procedure reaching a threshold established by an institutional policy, in alignment with standards or regulatory requirements^59^, ^60^ or for other quality assurance needs. Methods for these estimates vary, as indicated in previous sections. The accuracy requirements for patient dose estimates have not been established and are often even unknown, even to the clinical physicist performing the estimate.

The application of existing radiobiological models and principles to FGI procedures requires the most precise dosimetry reasonably possible. Fortunately, advancements are being made as imaging equipment vendors and third‐party software developers continue to refine dose mapping techniques.^2^, ^4^, ^5^, ^18^, ^46^ These techniques increasingly account for variables such as patient size and geometric factors. However, caution must be used by clinical physicists to understand what is and what is not included in any software or other automated dose calculation method. Moving forward, it is hoped that future systems will integrate more advanced corrections to account for additional sources of uncertainty—or, at the very least, explicitly acknowledge which factors are or are not accounted for in the dose calculations.

Radiation kerma and dose maps are recommended elements of the IEC 60601‐2‐43:2022 standard for fluoroscopes intended for use in interventional procedures. As manufacturers gain compliance with the IEC recommendations, it is anticipated that more interventional systems will natively incorporate these advanced dose‐mapping capabilities and display them to the equipment operators during procedures, improving the accuracy of dose estimation and enhancing patient safety. Although only recommended at this point, hopefully future versions of the standard will require these maps to be provided as part of the basic safety of these systems.

Improving radiation dosimetry will require continuous improvement in both equipment and dosimetric practices. Enhanced dose mapping techniques that account for beam nonuniformity, improved accuracy of displayed radiation dose indices, and more detailed reporting through systems like the P‐RDSR will be essential in reducing uncertainty. These advancements will help refine patient dosimetry and improve our understanding of associated radiobiological effects and may allow for the application of more advanced radiation biology models. Ultimately, they may help mitigate unnecessary radiation risks and reduce unnecessary patient follow‐up, contributing to better patient outcomes and more efficient clinical tracking in FGIs.

## 6 CONCLUSION

The evolution of interventional fluoroscopes and FGI procedures has transformed medicine, enabling the treatment of diseases once considered untreatable or only treatable by exceedingly invasive surgical intervention. However, this progress can come at the cost of substantial patient radiation doses. While modern advancements like standardized dose indices and RDSRs have greatly improved our ability to acquire and track dose data, this review demonstrates that substantial and often overlooked uncertainties remain.

Medical physicists must be acutely aware of these limitations. The convenience of automated dose estimation software should not obscure the fact that these systems may not account for various factors, such as the anode heel effect, the use of tissue compensation filters, or patient and C‐arm motion. A deeper understanding of these inherent uncertainties is essential for accurately interpreting dose estimates and providing reliable risk assessments to clinicians and patients.

Ultimately, enhancing the accuracy of FGI patient dosimetry is foundational to improving our understanding of radiobiology and providing better patient risk estimates. It is imperative that we bridge the gap between technical capability and clinical application by establishing more robust standards and integrating advanced corrections into our systems. This will not only lead to better‐informed clinical practice and improved DRL values but will also help build and refine radiobiological models to better understand the underlying risks associated with expanded clinical and imaging capabilities.

## CONFLICT OF INTEREST STATEMENT

The authors declare no conflicts of interest.
